# Rabies in the 21^st^ Century

**DOI:** 10.1371/journal.pntd.0000591

**Published:** 2010-03-30

**Authors:** William H. Wunner, Deborah J. Briggs

**Affiliations:** 1 The Wistar Institute, Philadelphia, Pennsylvania, United States of America; 2 Global Alliance for Rabies Control, Edinburgh, Scotland, United Kingdom; New York University School of Medicine, United States of America

Why are 50,000–55,000 people dying from rabies worldwide each year, with 25,000–30,000 human deaths in India alone and over 3 billion people continuing to be at risk of rabies virus infection in over 100 countries in the 21^st^ century? These are astonishing numbers, particularly as they represent individuals, a large proportion of whom are children, who have been attacked or are likely to be attacked by rabid dogs, the main source of rabies virus infection that, as yet, has not been brought under control in many parts of the world. The number of human deaths and the circumstances by which these deaths continue to occur are extraordinary, with over 95% of rabies victims reported residing in Asia and Africa and nearly all victims of a rabid dog bite. Rabies has been part of the history of civilization for several millennia, rooted in its enzootic environment (animal host) and causing severe threats to public health across continents. Rabies and the symptoms it presents can hardly be ignored, yet it appears to be unduly neglected in some parts of the world, notably in Asia and Africa, where the spread of canine rabies is not under control and is far from being eliminated. In other parts of the world, largely in developed countries, where elimination of canine rabies has been achieved, there are models to be followed and lessons learned that will challenge epidemiologists and molecular virologists alike in the future as they apply new techniques to achieve the elimination of canine and human rabies worldwide.

Through the World Rabies Day (WRD) initiative (www.worldrabiesday.org), over 55 million people have received educational material about rabies prevention. Today, people from more than 85 countries are involved on all levels of society (government, medical and veterinary professionals, media, educators, and lay people), ready to take some action toward elimination of endemic rabies worldwide. Despite the many languages and different cultures involved and so little money to work with, the empowerment of people around the world to do something for their own communities and countries is what has made the WRD initiative successful. Educational materials have been created that are easily translated into different languages and distributed through electronic media, and with these materials people are becoming better educated about rabies prevention. People are learning that going to local healers for treatments that do not work, such as rubbing chili powder in wounds, incantations, and taking ineffective herbal medicines, is not the way to prevent rabies. Instead, people learn from the educational materials that the risk of exposure to rabies can be minimized and the disease can be prevented by using the right methods and treatments, and together these measures can make a difference in their own lives.

In several recent *PLoS Neglected Tropical Diseases* papers on rabies (2009–2010)—marking the third anniversary of World Rabies Day—scientists describe the situation of canine rabies control in developing countries, as well as various recent advances in the development of vaccines and treatments for rabies that will contribute to the elimination of human deaths from rabies. Reduction in the number of human deaths due to rabies has to begin with the elimination of canine rabies in these countries. The feasibility of eliminating canine rabies in Africa [1] is predicated on the understanding and counteracting of the many reasons that canine rabies control has failed in Africa. It is interesting that the authors conclude that there are no reasons, nor any insurmountable problems, that would prevent canine rabies control from being achievable in most of Africa. In one of the papers, the authors state, “elimination of canine rabies is epidemiologically and practically feasible through mass vaccination of domestic dogs; and that domestic dog vaccination provides a cost-effective approach to the prevention and elimination of human rabies deaths” [1].

The lessons learned from epidemiological studies and the development of spatial models forecasting animal susceptibilities to enzootic rabies might be used for prevention and control of canine rabies, as well as other emerging zoonoses, in rabies endemic areas of the world [2]. With better surveillance methods for predicting newly emerging rabies epizootics through an understanding of the spatial dynamics and actual spread of enzootic rabies by any given host species, it would seem that the application of subsequent interventions, such as a vaccination program, can be improved. Conducting coordinated wildlife rabies management programs, particularly those relying heavily on oral rabies vaccination strategies, requires substantial interjurisdictional collaboration. For example, recent advances in coordinated surveillance practices, referred to as “enhanced rabies surveillance” and involving search and control measures, have greatly facilitated detection of animal rabies cases in a number of border areas shared by Canada, Mexico, and the United States, and have led to definitive actions for controlling rabies in strategically key areas [3]. At the basis of rabies control strategies are the validated diagnostic tests for rabies virus or a lyssavirus variant (there are at least six lyssavirus genotypes in addition to the rabies virus genotype). The molecular tools, which are readily accessible and easily used for detection of viral RNA and even species-specific viral RNA sequences, are becoming more widely accepted for the diagnosis of rabies [4]. Above all, in developing countries, diagnostic laboratories must operate under the precept that the lower the cost and the greater the “artlessness” of the molecular diagnostic tool, the better the chance that modern diagnostic techniques will be used for the diagnosis of rabies. In their review [4], Fooks et al. describe some of the latest developments and diagnostic techniques for determining the presence of rabies virus or nucleic acid in diagnostic samples. The authors write, “In the 21^st^ century, it is expected that diagnostic virology techniques for high throughput rabies virus detection will progress rapidly towards the use of molecular diagnostics replacing more conventional testing techniques such as virus isolation and histopathology” [4].

Vaccination is the most effective method of pre- or post-exposure medical intervention against rabies. What is clearly different and crucial in the case of human rabies is that, compared to any other human or animal pathogen-induced disease, it is the most severe of all infectious diseases, to the point of being almost invariably fatal. Fortunately, safe and efficacious commercially prepared cell culture-based vaccines are available to prevent rabies. The downside of most of these vaccines for use in the developing world is that they are too costly to produce and they have to be administered repeatedly—three times for pre-exposure vaccination and four or five times for post-exposure prophylaxis (PEP). In addition, for the more severe exposures, a regimen of vaccine combined with inoculations of rabies immunoglobulin (RIG) of human (HRIG) or equine (ERIG) origin for passive protection is required [5]. What the countries of the developing world need instead is an exceedingly inexpensive (compared to the cost of current conventional vaccines), safe vaccine that will provide sustained protection, preferably from a single dose. It is the goal of scientists in the 21^st^ century to develop novel rabies vaccines for use in humans. Many of these novel vaccines have advantages and disadvantages in their current stages of development compared to the cell culture-based vaccines currently in clinical use [5]. Nevertheless, advances are being made with the use of recombinant and reverse genetics techniques to construct highly immunogenic (immunogenicity that is increased by insertion of one or more additional glycoprotein genes), fully attenuated (for safety) rabies virus vaccines that can be scaled up in cell culture systems at low cost. Thus far, several of these novel vaccine candidates, which have gone through preclinical testing in laboratory animals, show considerable promise for achieving protection in mice with a single moderate dose of the vaccine. They are, however, several years away from possible acceptance for use in humans. Just as rabies vaccines are going through a revolutionary development process, new types of adjuvants are being evaluated, which, at least in mice, have a dose-sparing effect [5]. Whether transgenic plants will eventually be a suitable host and provide sizeable crops for production of subunit proteins of rabies virus as edible or nonedible vaccines is also under investigation, as are naked DNA vaccines. Plasmid DNA or replicon-based self-replicating DNA vaccines have the clear advantage of being easy to generate and produce in large amounts. They are likely to be more cost-effective to produce than purified subunit vaccines that require mammalian cell culture systems for production, but their effectiveness (immunogenicity and ability to protect) against rabies in humans has not yet been fully determined. The slow onset of an immune response to the transgene product of a DNA vaccine makes their usefulness for PEP doubtful. Other candidates in the arsenal of novel rabies vaccines currently under investigation include the recombinant heterologous viral vectors, such as various types of poxvirus and adenovirus vectors. Two novel recombinant poxviral vector vaccines, one using the Copenhagen strain of vaccinia virus (V-RG) and the other the canarypox virus (ALVAC), each expressing the rabies virus glycoprotein, are licensed and produced commercially for oral immunization of wildlife (raccoons, coyotes, or cats). The V-RG vaccine does not induce adequate protective immunity in other species such as skunks and dogs when administered orally, however, so alternative oral vaccines need to be identified to target these species, particularly dogs, since they serve as major reservoirs for rabies worldwide, especially in Asia and Africa. The modified vaccinia virus Ankara (MVA) has been considered a possible alternative, although preliminary observations suggest that when administered orally, it fails to elicit anamnestic immune responses in dogs and raccoons that have previously been vaccinated. Further work must continue in the 21^st^ century to find and develop these and other new-generation oral vaccines for animal species that are the reservoirs for rabies.

Considerable attention has been directed in recent years to the recombinant adenovirus (Ad)-based vectors that are derived from different human Ad serotypes and animal species serotypes for use as vaccines [5]. A problem with using human Ad serotypes arises, however, from the fact that adenoviruses are common pathogens in humans and it is highly probable that an individual who is immunized with a human Ad vector will already have neutralizing antibodies to the Ad vector. In such cases, the prevalence of neutralizing antibodies in the host will weaken the immune response to the human Ad vector and the expressed pathogen-specific gene product encoded in the vector when given as a vaccine. Therefore, an alternative Ad vector derived from an animal species (unlikely to have infected humans) may be considered a more suitable vaccine carrier to protect humans from rabies than the more common human Ad serotypes. Accordingly, several alternative vaccine vectors derived from chimpanzee adenoviruses (AdC) are being tested, since most humans are not likely to have neutralizing antibodies to the AdC serotypes before immunization with the AdC vectors as vaccines [5]. Whether these novel vaccine vectors will prove more suitable for large-scale, low-cost prophylactic vaccination in resource-poor countries and provide adequate PEP (after exposure to a rabid animal) with fewer doses of the vaccine than are required with conventional vaccine regimens remains to be determined with further investigation.

Knowing whether a person who received a rabies vaccine will be protected from a potentially lethal rabies virus challenge is sometimes dependent solely on the laboratory assessment of circulating antibodies that the person developed following immunization. The selection of the appropriate assay(s) to assess an individual's antibody titer and the validation of the assay method used therefore become extremely critical. For a fatal disease like rabies, these considerations, though often complex, are of paramount importance. To adhere to the principle that the appropriate assay will be used is especially significant when the results from such assays serve as a surrogate marker for the expected level of disease prevention. Laboratories that provide these important diagnostic services throughout the world need to pay special attention to the standardization and validation of the methods they use and should require proficiency testing, training, and certification of staff involved in performing such tests [6].

One of the significant contributing factors to the unacceptably high death rate from human rabies in the developing world is the severe shortage to nonexistence of the recommended components of PEP, HRIG, or ERIG. In the PEP treatment of patients, it is critical that RIG be administered with the initial dose of vaccine following a bite from a rabid animal to provide passive immunity to neutralize virus at the wound site until active immunity stimulated by the vaccine takes over. With the increased demand for post-exposure treatment in the developing world, as in developed countries, the world's supply has failed to provide these needed biological components. Replacement of HRIG and ERIG with cheaper and efficacious alternative biologicals for treatment of rabies in humans is therefore a high priority. Accordingly, cocktails of mouse monoclonal antibodies (MuMAbs) and human monoclonal antibodies (HuMAbs) are presently being assessed as replacements for RIG [7], [8].

Development of antiviral biologics other than MAbs for the therapeutic intervention of human rabies has not received as much attention as is warranted, mainly because it has been so difficult to target a virus that infects almost exclusively neurons and replicates predominantly in neurons of the central nervous system (CNS). Recently, scientists started to investigate one of the major characteristics of pathogenic rabies virus: its ability to suppress responses of the immune system [9]. Being able to cross the BBB into the CNS is key to having rabies virus–specific antibody-producing B cells in the CNS that are capable of neutralizing rabies virus, and immune effector T cells and molecules to clear rabies virus from the CNS tissues. It is well known that the neuropathogenesis of virulent, and not attenuated, rabies virus is associated with its ability to prevent delivery of immune effector T cells and B cells across the BBB to control virus replication and clear virus from the CNS [10]. The possibility that superinfection with an attenuated (live, nonpathogenic) rabies virus, as a vaccine, could be a new strategy for the treatment of a pathogenic rabies virus infection after the virus has reached the CNS and signs of the disease have appeared is intriguing [10].

The public worldwide continues to be at risk of exposure to rabies, whether it be in developing countries where control of canine rabies has been largely neglected or in developed countries where the potential risk from enzootic rabies is primarily from exposure to a variety of wildlife animal species. Communicating that risk is a matter of understanding communication principles and stakeholder responsibilities. The 74-country World Rabies Day initiative launched in 2007 brought urgent attention to the need to address the global threat of rabies more strategically worldwide and for each country to act domestically. The excellent example of the successful canine rabies elimination within the United States that was announced by the Centers for Disease Control and Prevention (CDC) was brought about over many decades by local, state, and federal public health authorities [11], [12]. Also, collaboration with the US Department of Agriculture, which has been responsible for oral rabies vaccination of wildlife in the US, represents an ongoing strategic activity to reduce or eliminate enzootic rabies from wildlife reservoirs. Many lessons can be learned from the actions taken by local, state, and federal authorities in the US in their efforts to devise “risk communication” strategies. It is the hope of scientists, public authorities, and veterinary and medical professionals worldwide that it will not take another century before one of the most severe and often-neglected diseases threatening animals and humans alike is eliminated.
